# Differential risk and clinical characteristics of placenta accreta spectrum in twin and singleton pregnancies: implications for perinatal outcomes

**DOI:** 10.7189/jogh.15.04252

**Published:** 2025-08-22

**Authors:** Wei-Zhen Tang, Kang-Jin Huang, Xia Li, Qin-Yu Cai, Ying-Xiong Wang, Hong-Yu Xu, Li Wen, Lan Wang, Tai-Hang Liu

**Affiliations:** 1 Department of Obstetrics and Gynecology, Women and Children’s Hospital of Chongqing Medical University, Chongqing, China; 2 Department of Bioinformatics, School of Basic Medical Sciences, Chongqing Medical University, Chongqing, China; 3 The Joint International Research Laboratory of Reproduction and Development, Chongqing Medical University, Chongqing, China

## Abstract

**Background:**

This study compares the prevalence of placenta accreta in singleton and twin pregnancies and examines its impact on adverse perinatal outcomes, exploring whether twin gestation increases the risk of poor outcomes in placenta accreta cases.

**Methods:**

A multivariate logistic regression analysis assessed the link between twin pregnancy and placenta accreta, comparing associated adverse perinatal outcomes in twin *vs*. singleton pregnancies. Stratified and interaction analyses explored clinical characteristics' relationship with placenta accreta. The Restrictive Cubic Spline (RCS) model evaluated the impact of placenta accreta on caesarean section and postpartum haemorrhage at different gestational ages. A comparative analysis examined clinical features and perinatal outcomes between twin and singleton pregnancies with placenta accreta. Finally, mediation analysis was used to determine if placenta accreta mediates the effect of twin gestation on caesarean section and postpartum haemorrhage.

**Results:**

In a large cohort study of 16 908 pregnancies, including both twin and singleton pregnancies, conducted in Chongqing, China, the risk of placenta accreta increased by 51% in twin gestations, with haemorrhagic placenta accreta rising by 133%. This condition significantly heightened the risk of adverse perinatal outcomes in both singleton and twin pregnancies, with twin pregnancies exhibiting higher risks. In twins, the risk of preterm birth was 1.77 (95% confidence interval (CI) = 1.24, 2.52), caesarean section was 4.87 (95% CI = 3.00, 7.90), postpartum haemorrhage was 3.73 (95% CI = 1.95, 7.13), and uterine rupture was 26.42 (95% CI = 2.28, 306.63). Additionally, placenta accreta showed different interactions with various factors in both twin and singleton pregnancies, influencing distinct outcomes. Restricted Cubic Splines (RCS) model analysis indicated an increasing trend in the risk of caesarean section and postpartum haemorrhage associated with placenta accreta across all gestational ages in both singleton and twin gestations. In patients with placenta accreta, the risks of preterm birth, caesarean section, pelvic inflammatory disease, atonic postpartum haemorrhage, and premature rupture of membranes in twin gestations were 6.77, 2.39, 2.54, 5.84, and 2.93 times higher, respectively, than in singleton gestations. Finally, mediation causal analysis revealed that the effect of twin gestation on caesarean section included both a direct effect and an indirect effect mediated through placenta accreta. For postpartum haemorrhage, the effect of twin gestation was mediated through placenta accreta.

**Conclusions:**

Twin gestation, regardless of known risk factors, increases the risk of placenta accreta and adverse perinatal outcomes compared to singleton pregnancies. Antenatal interventions and delivery risk management are essential for twin pregnancies with placenta accreta.

Placenta accreta spectrum (PAS) disorders represent a grave maternal complication characterised by the abnormal adherence of the placental trophoblast to the myometrium [1]. The incidence of PAS varies among populations, typically ranging between 0.01 and 1.1% [2,3]. Given that the diagnosis of PAS primarily relies on histopathology, the true incidence remains elusive due to the lack of uniform diagnostic criteria. Patients with PAS face severe risks, including catastrophic haemorrhage, hysterectomy, organ damage, consumptive coagulopathy, and even maternal death [2,4,5]. With an accelerating incidence of PAS, there is a concurrent increase in caesarean section rates, particularly in resource-rich countries where they have surpassed 30% [6-8]. Caesarean deliveries, where the placenta physiologically fails to separate from the uterus, can lead to significant obstetric haemorrhage upon forced removal, potentially increasing morbidity and mortality risks [9,10]. Therefore, improving diagnostic accuracy and timely intervention for potential PAS is essential. Optimising the location and timing of delivery is also crucial to reduce maternal complications.

Despite numerous studies identifying risk factors for PAS, such as placenta previa, previous caesarean section, history of uterine surgery, and treatment with ART [11,12], the relative risk posed by twin gestation to PAS remains unclear. Theoretically, due to the larger surface area occupied by twin placentas in the lower uterine segment, there could be more abnormal trophoblastic invasion at the lower segment or previous caesarean scar sites. However, existing cohort study results on the relationship between twin gestation and PAS are markedly inconsistent. Miller et al. [13] reported that the risk of PAS in twin pregnancies was 2.5 times that of singleton pregnancies, while Guo et al. [14] found a 1.63-fold increased risk. Conversely, Matsuzaki et al. [5] reported a lower incidence of PAS in multiple gestations compared to singleton gestations (odds ratio (OR) = 0.73). Given the controversies in existing research, this retrospective cohort study aims to explore whether twin gestation is an independent risk factor for PAS and to compare adverse perinatal outcomes between PAS patients in twin *vs*. singleton pregnancies. Most existing studies rely on International Classification of Diseases (ICD) codes to diagnose PAS, but Jotwani et al. have pointed out that ICD-10 has relatively low accuracy in diagnosing PAS, which can lead to misdiagnosis [13,14]. To avoid this, our study uses pathologically or clinically confirmed cases, ensuring high diagnostic accuracy. Additionally, the strengths of our study include the use of a multi-year database from a renowned tertiary teaching hospital in Southwest China, ensuring the richness, continuity, and relevance of the data. Previous studies, such as those by Matsuzaki and Guo, did not exclude pregnancies with three or more foetuses, which could introduce confounding factors [5,14]. In contrast, our study focuses exclusively on twin and singleton pregnancies, excluding those with three or more foetuses to reduce confounding variables and allow for a more precise assessment of PAS risk. Furthermore, we will examine additional pregnancy outcomes, such as gestational diabetes mellitus (GDM), preeclampsia (PE), and intrahepatic cholestasis of pregnancy (ICP). Through mediation analysis, we will explore how placenta accreta influences primary outcomes in twin *vs*. singleton pregnancies. By comparing PAS risk and its impact on primary outcomes (such as caesarean section and postpartum haemorrhage) in twin *vs*. singleton pregnancies, this study aims to provide insights for early screening and intervention. Additionally, the study will analyse PAS characteristics under different gestational modalities, offering valuable data for clinicians and researchers to better identify and manage PAS risks. Ultimately, the findings will inform strategies for optimising PAS management and reducing adverse perinatal outcomes.

## METHODS

### Ethical approval

Our study conformed to the Strengthening the Reporting of Observational Studies in Epidemiology (STROBE) reporting guideline for cohort studies. The study was approved by the Women and Children’s Hospital of Chongqing Medical University (ID: 2022-011-01), and the requirement for informed consent was waived because the data were deidentified.

### Data collection

The individuals were identified from the institution's electronic medical record system at Women and Children’s Hospital of Chongqing Medical University, a tertiary teaching hospital in Southwest China, and their electronic records were extracted for relevant data, encompassing basic demographic information (maternal age, body mass index (BMI)) and obstetric history (previous Caesarean section). This ensures the reliability and representativeness of the data. These records were reviewed and data were extracted by trained medical professionals, ensuring the reliability and representativeness of the data. The current pregnancy characteristics considered included the use of ART, as well as pregnancy complications such as placenta previa, multiple gestation, anaemia, hypothyroidism, GDM, PE, ICP, and foetal growth restriction (FGR). Perinatal adverse outcomes included caesarean section, postpartum haemorrhage, premature delivery, pelvic inflammation, atonic postpartum haemorrhage, premature rupture of membranes, uterine rupture, umbilical cord entanglement, and foetal distress. Placental characteristics (*e.g*. racquet placenta, velaria placenta) and placental pathologies (*e.g*. placenta previa, placenta previa with bleeding, central placenta previa, marginal placenta previa, low-lying placenta) were also documented. The data were systematically compiled from electronic records, ensuring comprehensive inclusion of detailed demographic and clinical characteristics. Our results and variables are primarily based on strict pathological or clinical diagnoses The diagnostic criteria used for data inclusion were clearly defined and consistently applied throughout the review process.

### Study collection

This retrospective cohort study was conducted at the affiliated Women's and Children's Hospital of Chongqing Medical University in China. The study retrospectively included pregnant women who underwent regular prenatal check-ups and delivered between 2017 and 2022. The inclusion criteria were as follows:

(i) Maternal age ≥18;

(ii) Twin and singleton pregnancies.

The exclusion criteria were:

(i) Congenital malformations;

(ii) Missing data for key variables by more than 20%.

**Figure 1:**
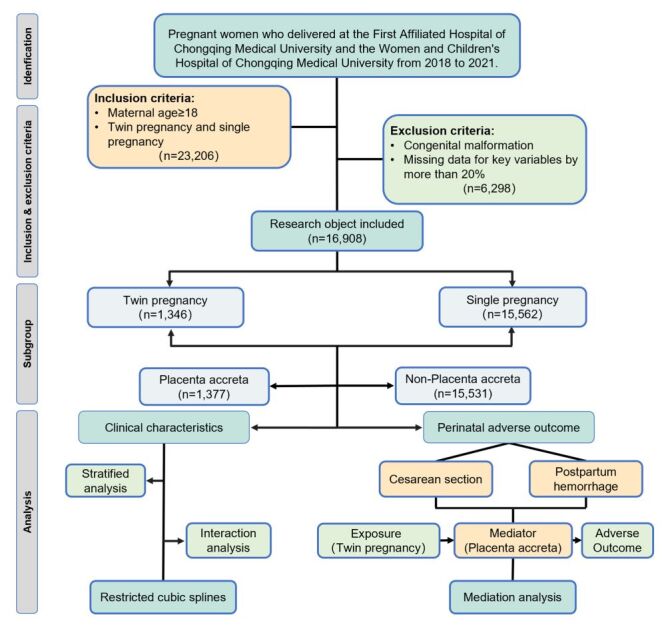
Flowchart of this retrospective cohort study.

A total of 23 206 pregnant women met the inclusion criteria. After applying the exclusion criteria, 16 908 cases were ultimately included in the study (**Figure 1**). Since existing studies have primarily focused on the differences in the incidence of placenta accreta between singleton and twin pregnancies, and research exploring the impact on pregnancy outcomes is relatively scarce, with no studies having performed a sample size calculation, we were unable to find reliable references to accurately calculate the sample size. Additionally, as an exploratory study, we believe that the sample size in this research is sufficient to support the preliminary analysis of the study. We adhered to the STROBE (Strengthening the Reporting of Observational Studies in Epidemiology) guidelines for cohort studies.

### Definition

All pregnancies managed at the centre underwent standardised and comprehensive placental evaluation. As recommended by published guidelines [15], pregnancies with a history of caesarean section and/or uterine surgery, low-lying placenta, and placenta previa were subjected to more stringent screening for PAS based on ultrasonographic findings. The specific ultrasonographic signs of PAS were consistent with those reported in previous guidelines [16,17]. Magnetic Resonance Imaging (MRI) was employed as an adjunct to ultrasound in cases where PAS was suspected to affect adjacent pelvic organs, posterior placenta, maternal obesity impeding the accuracy of ultrasound diagnosis, or when ultrasound assessment was indeterminate. The diagnostic criteria for PAS were as follows:

(i) Pathological criteria [18]: abnormal adherence of chorionic villi to the superficial or deep myometrium without intervening decidua, as diagnosed microscopically;

(ii) Clinical diagnostic criteria in accordance with the International Federation of Gynecology and Obstetrics (FIGO) guidelines [19].

### Primary outcome

In this study, caesarean section and postpartum haemorrhage were chosen as the primary perinatal adverse outcome measures. The reasons for selecting these outcomes are as follows: according to the American College of Obstetricians and Gynecologists (ACOG), caesarean hysterectomy is the primary and preferred treatment for women with PAS, making caesarean section a key outcome for evaluating PAS patients [20,21]. Additionally, previous studies have shown a close association between invasive placenta and postpartum haemorrhage [22], with research indicating that up to 90% of women require blood transfusion postpartum [23]. This further supports the selection of postpartum haemorrhage as another important outcome measure.

### Secondary outcomes

Secondary outcomes included premature delivery, pelvic inflammation, atonic postpartum haemorrhage, premature rupture of membranes, uterine rupture, umbilical cord entanglement, and foetal distress. These outcomes were all of concern in the previous PAS study [24–26].

### Statistical analysis

Missing data were addressed using multiple imputation techniques, assuming that the data were missing at random (MAR). This assumption implies that the probability of missing data depends on observed data but not on unobserved data. The imputation process was carried out with multiple imputed data sets to ensure robust and unbiased estimates in the analysis. Continuous variables were compared using the independent Student's *t* test or Mann-Whitney U test, depending on the normality assumption, and were reported as mean ± standard deviation (SD) or median (interquartile range, IQR). Categorical variables were presented as numbers and percentages and compared using the χ^2^ test or Fisher exact test. To assess the risk of placenta accreta occurrence in twin pregnancies, univariate and multivariate logistic regression analyses were performed. In Model 2, adjustments were made for maternal age, pre-pregnancy BMI, nulliparity, primigravida, history of caesarean section and alcoholism. Model 3 further adjusted for placental morphology (such as racquet placenta and velaria placenta) and pregnancy complications (including GDM, PE, ICP, and FGR). Finally, Model 4 included adjustments for assisted reproductive technology (ART). The selection of covariates in our study was based on two main factors. First, we selected covariates based on baseline differences. Second, we adjusted for covariates that were associated with the impact of singleton *vs*. twin placenta accreta on pregnancy outcomes in previous studies [5,13,14,27,28]. Additionally, we further adjusted for covariates that were not considered in previous studies but which we believe may influence pregnancy outcomes, such as placental morphology and FGR [14,27,29]. This approach was taken to enhance the reliability and credibility of our analysis results. The variance inflation factor (VIF) was used to assess multicollinearity between variables in each model, with VIF values for all variables being below 10, indicating no significant multicollinearity issues. Additionally, propensity score matching was employed to eliminate baseline differences between twin and singleton pregnancies, with matching ratios of 1:1, 1:2, and 1:3. This approach was used to assess the true impact of twin pregnancies on the occurrence of placenta accreta and placenta accreta with bleeding. To explore the risks of perinatal adverse outcomes in twin and singleton pregnancies with placenta accreta, as well as the influence of twin pregnancies on perinatal outcomes within the placenta accreta cohort, eight subgroup analyses were conducted for the primary outcomes of caesarean section and postpartum haemorrhage. These analyses examined heterogeneity within clinically relevant subgroups, including age (advanced age >35 *vs*. non-advanced age ≤35), use of ART, history of caesarean section, placenta previa, anaemia, hypothyroidism, PE, and ICP. The selection of these factors for stratified analysis is based on the fact that numerous studies have demonstrated they are risk factors for placenta accreta [13,30,31]. These factors may alter the strength of the association between singleton and twin placenta accreta and postpartum haemorrhage as well as caesarean section. The impact of placenta accreta on the main outcomes within each subgroup was evaluated using generalized linear models (GLM) for both twin and singleton pregnancies. Interaction effects between placenta accreta and each subgroup variable were tested by including interaction terms in the GLM. Additionally, RCS were employed to examine the nonlinear relationship between gestational weeks at delivery and both caesarean section and postpartum haemorrhage outcomes. This analysis was conducted separately for twin and singleton pregnancies, with and without placenta accreta, to observe trends in ORs and the overlap of confidence intervals for the risk of placenta accreta. Four knots were chosen based on clinical considerations and data distribution. The nonlinearity of the relationship was formally tested by comparing the spline model to a linear model using a likelihood ratio test. All covariates previously mentioned in Model 4 were adjusted for in this analysis, ensuring that the influence of potential confounders was accounted for when modelling the nonlinear relationship. The reference value for gestational weeks at delivery was the median gestational week. Furthermore, causal mediation analysis was performed using the ‘mediation’ package to estimate the effect of twin pregnancy on caesarean section and postpartum haemorrhage mediated through placenta accreta. In addition, since all analyses conducted in this study are exploratory, no adjustments were made for multiple testing. All statistical analyses were conducted using SPSS software version 26.0 (IBM Corp., Armonk, NY, USA) and *R*, version 4.2.3 (The R Foundation for Statistical Computing, Austria, Vienna). The level of statistical significance for all tests was set at a two-tailed *P*-value of <0.05.

### Mediation analysis

To calculate the mediation effect of placental implantation between twin pregnancies and caesarean section or postpartum haemorrhage, the hypothesised model is shown in Figure S1 in the **Online Supplementary Document**. In this model, X represents twin pregnancy, Y represents either caesarean section or postpartum haemorrhage, M represents placental implantation, and C represents a set of confounding factors. This study employs a counterfactual causal mediation analysis method, based on the theoretical framework of Imai et al. [32], which allows for the decomposition of the total effect (TE) into the Average Direct Effect (ADE) and Average Causal Mediation Effect (ACME), corresponding to Natural Direct Effect (NDE) and Natural Indirect Effect (NIE). The calculation formulas for the effect values are provided in article [33]. This method is suitable for mediation models with binary mediator or outcome variables and can provide more accurate causal inference. First, the ‘lme4’ package in *R* software version 4.2.3 (R Foundation for Statistical Computing, Vienna, Austria) is used to fit a generalized linear mixed-effects model (GLMM) to account for the hierarchical structure of the data. Next, the ‘mediate’ function from the ‘mediation’ package is used to conduct the mediation effect analysis. In this analysis, we adjusted the confounding factors adjusted in Model 4. The analysis reports the following effect estimates: the total effect represents the overall impact of twin pregnancy on caesarean section and postpartum haemorrhage; the direct effect represents the impact of twin pregnancy on caesarean section or postpartum haemorrhage without the mediation of placental implantation; the indirect effect represents the impact of twin pregnancy on caesarean section or postpartum haemorrhage through placental implantation; and the proportion of mediation represents the ratio of the mediation effect to the total effect, calculated as Proportion Mediated = ACME/TE, which reflects the proportion of the total effect explained by the mediation path. The bootstrap confidence interval (bootstrap CI) is set to 95%, with 5000 bootstrap samples. If the 95% CI does not include zero, it indicates the presence of a significant mediation effect.

## RESULTS

In this study, after screening through inclusion and exclusion criteria, a total of 16 908 pregnancies were included in the final analysis (**Figure 1**). Of these, 1346 cases (7.96%) were twin pregnancies, while 15 562 cases (92.04%) were singleton pregnancies. The baseline characteristics analysis of the two groups revealed that the twin pregnancy group had significantly higher proportions of ART (22.14% *vs*. 1.92%, *P* < 0.001), nulliparity (86.33% *vs*. 63.66%, *P* < 0.001), primigravida (52.16% *vs*. 34.34%, *P* < 0.001), identifying these as potential risk factors (**Table 1**). In terms of age and pre-pregnancy BMI, twin pregnancies had a higher mean age and pre-pregnancy BMI (age = 30.93, interquartile range (IQR) = 28.61–33.44 *vs*. 30.58, IQR = 27.60–33.92, *P* = 0.006; pre-pregnancy BMI = 21.50, IQR = 20.03–23.53 *vs*. 21.23, IQR = 19.10–23.73, *P* < 0.001). Additionally, twin pregnancies had significantly higher risks of complications such as anaemia, FGR, and ICP (anaemia = 15.08% *vs*. 9.13%, *P* < 0.001; FGR = 7.65% *vs*. 1.38%; ICP = 16.94% *vs*. 3.93%). Regarding placental abnormalities, the rates of racquet placenta and velaria placenta were significantly higher in twin pregnancies (racquet placenta = 4.53% *vs*. 3.08%, *P* = 0.004; velaria placenta = 3.05% *vs*. 0.71%, *P* < 0.001), as were the risks for placenta accreta and placenta accreta with bleeding, and placenta previa with bleeding (placenta accreta = 13.00% *vs*. 7.72%, *P* < 0.001; placenta accreta with bleeding = 3.20% *vs*. 1.18%, *P* < 0.001; placenta previa with bleeding = 1.56% *vs*. 0.79%, *P* = 0.003). However, there were no statistically significant differences between the groups in terms of age at first menstruation, history of smoking, concurrent GDM, occurrence of placenta previa, including central, marginal, low-lying placenta, and placenta previa with accreta (**Table 1**).

**Table 1:** Demographic and clinical characteristics of women with singleton and twin pregnancies

Characteristics, n (%)	Twin pregnancy (n = 1346)	Singleton pregnancy (n = 15 562)	Statistic (χ^2^)	*P*-value
Age in years	30.93 (28.61,33.44)	30.58 (27.60,33.92)	−2.739	0.006*
PBMI, mdn (Q_1_, Q_3_)	21.50 (20.03,23.53)	21.23 (19.10,23.73)	−4.540	<0.001*
ART	298 (22.14)	298 (1.92)	1490.101	<0.001*
Nulliparity	1162 (86.33)	9907 (63.66)	281.577	<0.001*
Primigravida	702 (52.16)	5344 (34.34)	171.148	<0.001*
Age of first menstruation	12.00 (11.00,13.00)	12.00 (11.00,13.00)	−1.495	0.117
History of caesarean section	3 (0.22)	580 (3.73)	45.693	<0.001*
History of smoking	26 (1.94)	430 (2.78)	3.317	0.069
History of alcoholism	181 (13.59)	2463 (16.05)	5.570	0.018*
Uterine myoma	61 (4.53)	687 (4.42)	0.040	0.841
Anaemia	203 (15.08)	1421 (9.13)	50.523	<0.001*
Hypothyroidism	147 (10.92)	2275 (14.62)	13.802	<0.001*
GDM	493 (36.63)	5636 (36.22)	0.090	0.764
PE	253 (18.80)	627 (4.03)	547.581	<0.001*
FGR	103 (7.65)	215 (1.38)	263.977	<0.001*
ICP	228 (16.94)	611 (3.93)	444.833	<0.001*
Racquet placenta	61 (4.53)	479 (3.08)	8.470	0.004*
Velaria placenta	41 (3.05)	110 (0.71)	76.589	<0.001*
Placenta accreta	175 (13.00)	1202 (7.72)	46.125	<0.001*
Placenta accreta with bleeding	43 (3.20)	184 (1.18)	37.873	<0.001*
Placenta previa	43 (3.20)	396 (2.55)	2.070	0.150
Placenta previa with bleeding	21 (1.56)	123 (0.79)	8.694	0.003*
Central placenta previa	21 (1.56)	178 (1.14)	1.847	0.174
Marginal placenta previa	18 (1.34)	170 (1.09)	0.676	0.411
Low-lying placenta	8 (0.59)	137 (0.88)	1.192	0.275
Placenta previa with placenta accreta	18 (1.34)	144 (0.93)	2.216	0.137

ART – assisted reproductive technology, GDM – gestational diabetes mellitus, FGR – foetal growth restriction, ICP – intrahepatic cholestasis of pregnancy, mdn – median, PBMI – pre-pregnancy body mass index, PE – preeclampsia, Q_1_ – 1st quartile, Q_3_ – 3st quartile

*P < 0.05.

To explore the relative risk of placenta accreta and placenta accreta with bleeding in twin pregnancies, the unadjusted logistic regression model showed that twin pregnancies significantly increased the risk for placenta accreta and placenta accreta with bleeding (placenta accreta relative risk (RR) = 1.79, 95% CI = 1.51, 2.12; placenta accreta with bleeding RR = 2.76, 95% CI = 1.97, 3.86). After adjusting for maternal age, pre-pregnancy BMI, nulliparity, primigravida, and obstetric history (history of caesarean section, history of alcoholism), the association between twin pregnancy and placenta accreta remained significant, with a slight increase in RR to 1.87 (95% CI = 1.57, 2.23), and the risk for placenta accreta with bleeding also increased slightly, with an RR of 2.80 (95% CI = 1.98, 3.96) (**Table 2**). Further adjustments in the model for placental morphology (battledore placenta, velamentous cord insertion), placenta previa, and pregnancy complications (GDM, PE, ICP, FGR) increased the risk for placenta accreta and placenta accreta with bleeding, with RRs of 1.81 (95% CI = 1.51, 2.18) and 2.88 (95% CI = 2.01, 4.15), respectively. Finally, after considering the use of ART in the model, the risk of placenta accreta in twin pregnancies relative to singleton pregnancies decreased but remained significant, with an RR of 1.51 (95% CI = 1.24, 1.83); the risk for placenta accreta with bleeding also decreased slightly, with an RR of 2.33 (95% CI = 1.57, 3.45) (**Table 2**). In addition, we used propensity score matching to eliminate baseline differences between twin and singleton pregnancies, with matching ratios of 1:1, 1:2, and 1:3. Using this approach, we examined the true impact of twin pregnancies on the occurrence of placenta accreta and placenta accreta with bleeding. The results showed that regardless of the matching ratio, twin pregnancies significantly increased the risk of both placenta accreta and placenta accreta with bleeding. Detailed results can be found in Table S1 in the **Online Supplementary Document**.

**Table 2:** The association between twin gestation and placenta accreta

Variables	Model 1*	*P*-value	Model 2†	*P*-value	Model 3‡	*P*-value	Model 4§	*P*-value
Placenta accreta	1.79 (1.51, 2.12)	<0.001¶	1.87 (1.57, 2.23)	<0.001¶	1.81 (1.51, 2.18)	<0.001¶	1.51 (1.24, 1.83)	<0.001¶
Placenta accreta with bleeding	2.76 (1.97, 3.86)	<0.001¶	2.80 (1.98, 3.96)	<0.001¶	2.88 (2.01, 4.15)	<0.001¶	2.33 (1.57, 3.45)	<0.001¶

ART – assisted reproductive technology, GDM – gestational diabetes mellitus, FGR – foetal growth restriction, ICP – intrahepatic cholestasis of pregnancy, PBMI – pre-pregnancy body mass index, PE – preeclampsia

*Model 1: unadjusted, univariate analysis.

†Model 2: adjusted for maternal age, PBMI, nulliparity, primigravida, history of caesarean section, history of alcoholism.

‡Model 3: adjusted for racquet placenta, velaria placenta, placenta previa, GDM, PE, ICP, FGR plus variables adjusted in Model 2.

**§**Model 4: adjusted for ART plus variables adjusted in Model 3.

¶*P* < 0.05.

The comparative analysis of the clinical characteristics of placenta accreta and perinatal adverse outcomes between twin and singleton pregnancies revealed that twin pregnancies are more prone to placenta accreta under the conditions of advanced maternal age, use of ART, multiparity, absence of anaemia, foetal growth restriction, and the presence of placenta previa (including placenta previa with bleeding and central placenta previa) (Table S2 in the **Online Supplementary Document**). These factors constitute potential risk factors for placenta accreta in twin pregnancies. Regardless of singleton or twin gestation, women with placenta accreta had a significantly increased risk of preterm birth, caesarean section, postpartum haemorrhage, and antepartum uterine rupture compared to those without placenta accreta, with the relative risks being more pronounced in twin pregnancies. Specifically, the relative risks in twin pregnancies were 1.77 for preterm birth (95% CI = 1.24, 2.52), 4.87 for caesarean section (95% CI = 3.00, 7.90), 3.73 for postpartum haemorrhage (95% CI = 1.95, 7.13), and an alarmingly high 26.42 for antepartum uterine rupture (95% CI = 2.28, 306.63), which reflects a highly unstable estimate due to the wide confidence interval, likely resulting from sparse underlying data. In singleton pregnancies, these risks were 1.67 (95% CI = 1.32, 2.11), 2.67 (95% CI = 2.34, 3.04), 2.42 (95% CI = 1.87, 3.14), and 2.78 (95% CI = 1.78, 4.32), respectively. Additionally, in twin pregnancies with placenta accreta, the risk of atonic postpartum haemorrhage was significantly increased, with a relative risk of 4.03 (95% CI = 1.95, 8.35), whereas no significant difference was observed in singleton pregnancies. Notably, in singleton pregnancies, the risks of preterm birth, premature rupture of membranes, and foetal distress were significantly increased in women with placenta accreta, with relative risks of 1.64 (95% CI = 1.19, 2.26) and 1.34 (95% CI = 1.10, 1.63) respectively, while these complications were not significantly increased in twin pregnancies (**Table 3**).

**Table 3:** The impact of placenta accreta on the risk of adverse perinatal outcomes in twin *vs*. singleton pregnancies*

Variables	Twin pregnancy					Singleton pregnancy				
	**Placenta accreta**	**Non-placenta accreta**	***P*-value**	**Univariate analysis**	**Multivariate analysis**	**Placenta accreta**	**Non-placenta accreta**	***P*-value**	**Univariate analysis**	**Multivariate analysis**
Premature delivery	60 (34.29)	284 (24.25)	0.005†	1.63 (1.16, 2.29)	1.77 (1.24, 2.52)	88 (7.32)	619 (4.31)	<0.001†	1.75 (1.39, 2.21)	1.67 (1.32, 2.11)
Caesarean section	153 (87.43)	688 (58.75)	<0.001†	4.88 (3.08, 7.75)	4.87 (23.00, 7.90)	130 (10.82)	1313 (9.14)	0.045†	2.98 (2.63, 3.39)	2.67 (2.34, 3.04)
Pelvic inflammation	50 (28.57)	172 (14.69)	<0.001†	2.3 2(1.61, 3.35)	2.12 (1.44, 3.12)	143 (11.90)	742 (5.17)	<0.001†	2.48 (2.05, 3.00)	2.04 (1.68, 2.49)
Postpartum haemorrhage	17 (9.71)	31 (2.65)	<0.001†	3.96 (2.14, 7.32)	3.73 (1.95, 7.13)	77 (6.41)	402 (2.80)	<0.001†	2.38 (1.85, 3.06)	2.422 (1.87, 3.14)
Atonic postpartum haemorrhage	12 (6.86)	21 (1.79)	<0.001†	4.03 (1.95, 8.35)	3.99 (1.85, 8.63)	16 (1.33)	195 (1.36)	0.938	0.98 (0.59, 1.64)	0.99 (0.58, 1.68)
Premature rupture of membranes	20 (11.43)	117 (9.99)	0.558	1.16 (0.70, 1.92)	1.12 (0.66, 1.89)	45 (3.74)	328 (2.28)	0.001†	1.66 (1.21, 2.29)	1.64 (1.19, 2.26)
Uterine rupture	3 (1.71)	2 (0.17)	0.002†	10.20 (1.69, 61.45)	26.42 (2.28, 306.63)	26 (2.16)	104 (0.72)	<0.001†	3.03 (1.96, 4.68)	2.78 (1.78, 4.32)
Umbilical entanglement	36 (20.57)	207 (17.68)	0.353	1.21 (0.81, 1.79)	1.14 (0.75, 1.71)	308 (25.62)	3822 (26.62)	0.454	0.95 (0.83, 1.09)	0.97 (0.85, 1.11)
Foetal distress	6 (3.43)	26 (2.22)	0.328	1.56 (0.63, 3.85)	1.80 (0.70, 4.62)	130 (10.82)	1313 (9.14)	0.055	1.21 (0.99, 1.458)	1.34 (1.10, 1.63)

ART – assisted reproductive technology, GDM – gestational diabetes mellitus, FGR – foetal growth restriction, ICP – intrahepatic cholestasis of pregnancy, PBMI – pre-pregnancy body mass index, PE – preeclampsia

*aOR adjusted for maternal age, PBMI, nulliparity, primigravida, history of caesarean section, history of alcoholism, racquet placenta, velaria placenta, placenta previa, GDM, PE, ICP, FGR, ART.

†*P* < 0.05.

Further stratified analyses were conducted for singleton and twin pregnancies focusing on the two primary outcomes: caesarean section and postpartum haemorrhage. Regarding caesarean section, placenta accreta significantly increased the risk of caesarean section in both singleton and twin pregnancies, irrespective of advanced maternal age, presence of placenta previa, coexisting PE, and history of caesarean section. In singleton pregnancies, placenta accreta significantly increased the risk of caesarean section among women who underwent ART as well as those with anaemia, hypothyroidism, and ICP. However, in twin pregnancies, placenta accreta significantly increased the risk of caesarean section only in women who did not undergo ART and did not have the aforementioned conditions. Due to insufficient sample sizes after stratification, particularly in the subgroup of twin pregnancies with a history of caesarean section, we were unable to conduct a detailed analysis in this group. Consequently, significant associations between placenta accreta and caesarean section were only identified in the subgroup of twin pregnancies without a prior caesarean section.

For postpartum haemorrhage, placenta accreta significantly increased the risk in both singleton and twin pregnancies without concomitant preeclampsia or pregnancy-related ICP. In twin pregnancies, placenta accreta significantly increased the risk of postpartum haemorrhage regardless of advanced maternal age or the use of ART; however, in singleton pregnancies, this increased risk was significant only among women who were not of advanced age and had not undergone ART. Due to a lack of cases of postpartum haemorrhage in twin pregnancies with a history of caesarean section, placenta previa, anaemia, and hypothyroidism, the study only identified a significant increase in the risk of postpartum haemorrhage in twin pregnancies with placenta accreta without placenta previa, anaemia, and hypothyroidism. In singleton pregnancies, placenta accreta not only significantly increased the risk of postpartum haemorrhage in cases without placenta previa but also significantly elevated the risk irrespective of the presence or absence of anaemia and hypothyroidism.

Further product interaction analyses examined the interplay between placenta accreta and maternal characteristics among singleton and twin pregnancies. For the outcome of caesarean section, a significant interaction between placenta accreta and the use of ART was observed in both twin (*p*-interaction = 0.027) and singleton pregnancies (*p*-interaction = 0.013). In twin pregnancies, significant interactions were also observed between placenta accreta and both maternal age and ICP (age, *p*-interaction = 0.026; ICP, *p*-interaction = 0.001). Regarding postpartum haemorrhage, in singleton pregnancies, significant interactions were found between placenta accreta and both maternal age and anaemia status (age, *p*-interaction = 0.021; anaemia, *p*-interaction <0.001), whereas no significant interaction effects were observed in twin pregnancies. These findings highlight the pivotal role of maternal characteristics in modulating the perinatal outcome sensitivity to placenta accreta and suggest differential patterns of impact by placenta accreta in singleton and twin pregnancies (Table S3 in the **Online Supplementary Document**).

**Figure 2:**
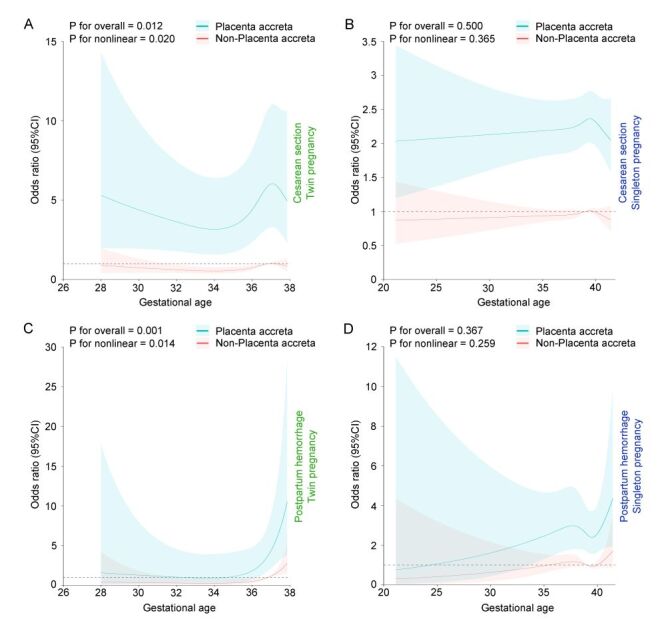
The association between the presence of placenta accreta and the odds ratios for adverse perinatal outcomes in twin *vs*. singleton pregnancies, as modelled by restricted cubic splines. **Panel A.** Caesarean section in twin pregnancies. **Panel B.** Caesarean section in singleton pregnancies. **Panel C.** Postpartum haemorrhage in twin pregnancies. **Panel D.** Postpartum haemorrhage in singleton pregnancies. The reference level for ORs is the median gestational week at delivery. The reference line is at Y = 1. These curves are adjusted for maternal age, PBMI, nulliparity, primigravida, the history of caesarean section, history of alcoholism, racquet placenta, velaria placenta, placenta previa, GDM, PE, ICP, FGR, ART. ART – assisted reproductive technology, GDM – gestational diabetes mellitus, FGR – foetal growth restriction, ICP – intrahepatic cholestasis of pregnancy, PBMI – pre-pregnancy body mass index, PE – preeclampsia.

We used restricted cubic splines to analyse the risk of caesarean section and postpartum haemorrhage in twin and singleton pregnancies with and without placenta accreta across various gestational weeks, to explore the risk of significant perinatal adverse outcomes. The analysis revealed that in twin pregnancies, gestational age significantly influenced the risk of caesarean section (*P* = 0.012) and exhibited a significant nonlinear relationship (*P* for nonlinearity = 0.020) (**Figure 2**, Panel A). However, gestational age did not significantly affect caesarean section risk in singleton pregnancies (*P* = 0.500), with no significant nonlinearity detected (*P* for nonlinearity = 0.365) (**Figure 2**, Panel B). Notably, in both singleton and twin pregnancies, women with placenta accreta exhibited a higher risk of caesarean section across all gestational ages, with a particularly significant increased risk at different gestational weeks in twin pregnancies compared to those without accreta. In singleton pregnancies, despite overlapping confidence intervals prior to 24 weeks due to small sample sizes, placenta accreta significantly increased the risk of caesarean section in subsequent gestational weeks. For postpartum haemorrhage, the analysis indicated that in twin pregnancies, gestational age also significantly affected the risk (*P* = 0.001) and a nonlinear relationship was present (*P* for nonlinearity = 0.014) (**Figure 2**, Panel C). However, there was no significant relationship between the risk of Postpartum haemorrhage and gestational age in singleton pregnancies (*P* = 0.367), with no significant nonlinearity observed (*P* for nonlinearity = 0.259) (**Figure 2**, Panel D). Nonetheless, women with placenta accreta in both singleton and twin pregnancies had a higher risk of postpartum haemorrhage, especially in twin pregnancies where the risk between 36–38 weeks was significantly higher than in those without accreta.

The investigation continued to examine the clinical characteristics of placenta accreta in singleton and twin pregnancies, as well as the impact of singleton *vs*. twin gestations on the occurrence of adverse perinatal outcomes in pregnancies complicated by placenta accreta. The results indicated that although there was no significant difference in the distribution of placenta previa types (including central, marginal, and low-lying placenta previa) between twin pregnancies with placenta accreta and singletons, twin gestations had a significantly higher risk of maternal complications, particularly PE and ICP (PE = 20.57% *vs*. 6.57%, *P* < 0.001; ICP = 17.14% *vs*. 3.16%, *P* < 0.001). Additionally, twin pregnancies with placenta accreta had a significantly higher proportion of velaria placenta and placenta previa with bleeding compared to singletons (velaria placenta = 4.57% *vs*. 1.58%, *P* = 0.008; placenta previa with bleeding = 5.71% *vs*. 2.25%, *P* = 0.008) (Table S4 in the **Online Supplementary Document**). When comparing adverse perinatal outcomes in placenta accreta cases between singleton and twin pregnancies, the data revealed that twins had higher rates of premature delivery, caesarean section, pelvic inflammation, atonic postpartum haemorrhage, and premature rupture of membranes (premature delivery = 34.29% *vs*. 7.32%, *P* < 0.001; caesarean section = 87.43% *vs*. 69.63%, *P* < 0.001; pelvic inflammation = 28.57% *vs*. 11.90%, *P* < 0.001; atonic postpartum haemorrhage = 6.86% *vs*. 1.33%, *P* < 0.001; premature rupture of membranes = 11.43% *vs*. 3.74%, *P* < 0.001). Conversely, the incidence of foetal distress was significantly lower in twin pregnancies compared to singletons (3.43% *vs*. 10.82%, *P* = 0.002). After adjusting for potential confounders, multivariable logistic regression analysis further confirmed these findings. Compared with singleton pregnancies, the adjusted relative risks for premature delivery, caesarean section, pelvic inflammation, atonic postpartum haemorrhage, and premature rupture of membranes in twin pregnancies with placenta accreta were 6.77 (95% CI = 4.41, 10.39), 2.39 (95% CI = 1.46, 3.91), 2.54 (95% CI = 1.68, 3.83), 5.84 (95% CI = 2.41, 14.17), and 2.93 (95% CI = 1.60, 5.37), respectively. The adjusted relative risk for foetal distress was 0.22 (95% CI = 0.09, 0.52) (Table S5 in the **Online Supplementary Document**). These results suggest that twin pregnancies with placenta accreta are at a higher risk of adverse perinatal outcomes compared to singleton pregnancies.

**Figure 3:**
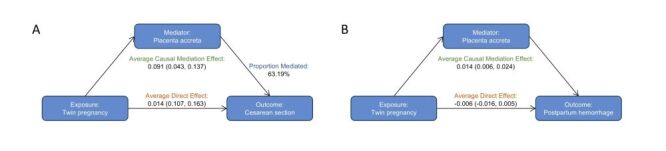
The mediation effects of placenta accreta on the relationship between twin pregnancy and caesarean section or postpartum haemorrhage. **Panel A.** Caesarean section. **Panel B.** Postpartum haemorrhage. Adjusted for maternal age, PBMI, nulliparity, primigravida, the history of caesarean section, history of alcoholism, racquet placenta, velaria placenta, placenta previa, GDM, PE, ICP, FGR, ART. The proportion mediated represents the proportion of the total effect that is accounted for by the mediation effect. It is calculated as proportion mediated = ACME/TE. This proportion reflects the extent to which the total effect is explained through the mediating pathway. ACME – Average Causal Mediation Effect, ART – assisted reproductive technology, GDM – gestational diabetes mellitus, FGR – foetal growth restriction, ICP – intrahepatic cholestasis of pregnancy, PBMI – pre-pregnancy body mass index, PE – preeclampsia, TE – total effect.

To further investigate the potential mechanisms by which placenta accreta may mediate the impact of twin pregnancies on significant adverse perinatal outcomes, results from the mediation model, controlling for confounding factors, suggested that the status of placenta accreta partially explains how twin pregnancies affect the risks of caesarean section and postpartum haemorrhage. Specifically, for caesarean section, the total effect of twin pregnancies on caesarean section was 0.144 (95% CI = 0.115, 0.172, *P* < 0.001), with a significant direct effect of 0.135 (95% CI = 0.107, 0.163, *P* < 0.001) and a significant indirect effect mediated by placenta accreta of 0.091 (95% CI = 0.043, 0.137, *P* < 0.001) (**Figure 3**, Panel A). Placenta accreta mediated 63.19% of the association between twin pregnancies and caesarean section (Table S6 in the **Online Supplementary Document**). This result reveals the crucial role of placenta accreta in how twin pregnancies influence the risk of caesarean section, indicating that twin pregnancies not only directly increase the likelihood of caesarean section but may also indirectly increase the risk through an increased risk of placenta accreta. For postpartum haemorrhage, the situation is different. The total and direct effects of twin pregnancies on postpartum haemorrhage were not significant (total effect = −0.004, 95% CI = −0.014, 0.006, *P* = 0.411; direct effect = −0.006, 95% CI = −0.016, 0.005, *P* = 0.285) (**Figure 3**, Panel B), but the indirect effect through placenta accreta was significant at 0.014 (95% CI = 0.006, 0.024, *P* < 0.001) (Table S7 in the **Online Supplementary Document**). This implies that the impact of twin pregnancies on postpartum haemorrhage is entirely realised through the mediating pathway of placenta accreta, indicating that twin pregnancies do not directly increase the risk of postpartum haemorrhage but indirectly affect its occurrence by increasing the risk of placenta accreta. This finding suggests that the association between postpartum haemorrhage and twin pregnancies is not a direct effect but is mediated through the variable of placenta accreta.

## DISCUSSION

In this study, twin pregnancies were found to independently increase the risk of placenta accreta by 1.51-fold compared to singleton pregnancies, consistent with findings by Miller et al. and Guo et al. [13,14]. Jauniaux et al. [34] speculated that the rise in iatrogenic factors such as caesarean section and ART might contribute to the increased incidence of placental implantation disorders, including PAS, which could directly or indirectly affect the integrity of the endometrial function. ART is known to be a risk factor for both the spectrum of placental implantation abnormalities and twin pregnancies [35–38], which partly explains the higher risk of placental implantation abnormalities in women with twin pregnancies. Notably, our analysis indicates that the findings hold true even when the impact of ART is not considered. The current literature does not fully report the adjusted association between ART and twin pregnancies, as well as ART and the spectrum of placental implantation disorders, with respect to age and related confounding factors. Our bias analysis, which controlled for ART, found that the risk of placenta accreta in twin pregnancies exists independently but is attenuated, consistent with the study by Miller et al. [13], suggesting that ART may play a significant role. Furthermore, previous studies have also indicated that in twin pregnancies, the placenta may be more likely to implant on a prior caesarean section scar, thereby initiating placenta accreta. Nonetheless, the specific mechanisms linking twin pregnancies to placenta accreta require further exploration. Clinicians should be aware of the increased risk of placenta accreta in twin pregnancies and be adequately prepared for potential scenarios, especially the potential causes of postpartum haemorrhage.

A substantial body of research has confirmed that placenta accreta increases the risk of adverse perinatal outcomes such as caesarean section, postpartum haemorrhage, hysterectomy, and maternal mortality [39]. Our study confirms that placenta accreta significantly increases the risks of postpartum haemorrhage and caesarean section in both singleton and twin pregnancies. It also raises the risks of preterm birth and uterine rupture, with these risks being higher in twin pregnancies. Particularly in twin pregnancies with placenta accreta, women are more susceptible to atonic postpartum haemorrhage. RCS analysis showed that women with placenta accreta in both singleton and twin pregnancies had a higher trend of undergoing caesarean section and experiencing postpartum haemorrhage across all gestational weeks. The risk for caesarean section was notably higher in twin pregnancies with placenta accreta at all gestational weeks compared to those without. In singleton pregnancies, there may be an overlap in confidence intervals due to fewer deliveries before 24 weeks, but the risk of caesarean section with placenta accreta is significantly higher in subsequent gestational weeks. For postpartum haemorrhage, twin pregnancies with placenta accreta had a significantly higher risk of bleeding between 36 and 38 weeks compared to those without, whereas singleton pregnancies with placenta accreta not only had a higher risk in this gestational window but also across a broader range of weeks.

There is ample evidence supporting that prenatal detection of the placenta accreta spectrum and management by a multidisciplinary team can reduce severe perinatal adverse outcomes. However, prenatal detection of placenta accreta remains challenging in twin pregnancies, even by experienced teams [40–42], particularly when a larger proportion of placentas are posteriorly located, considering the acoustic shadowing from the foetal parts and the fact that invasive placentas are not anteriorly positioned, making prenatal detection more difficult. Given that the relative risk of adverse perinatal outcomes is higher in twin pregnancies with placenta accreta compared to singleton pregnancies, this may reflect a lack of prenatal diagnosis and early intervention in twin pregnancies with placenta accreta. Further prospective research should address the differences in prenatal diagnosis of placenta accreta between singleton and twin pregnancies and whether it affects the incidence of complications. This underscores the importance of understanding the risk factors for placenta accreta in twin pregnancies. Our study identifies advanced maternal age, use of ART, multiparity, non-anaemic status, absence of foetal growth restriction, and the presence of anterior placenta (including those with bleeding and central placenta previa) as potential risk factors for placenta accreta in twin pregnancies. Moreover, these characteristics may play a key role in modulating the sensitivity of perinatal outcomes to placenta accreta. Regarding caesarean section, there is a significant interaction between placenta accreta and ART in both twin and singleton pregnancies; in twin pregnancies, there is also a significant interaction with age and ICP, while in singleton pregnancies, there is a significant interaction with the status of placenta previa. For Postpartum haemorrhage, in singleton pregnancies, there is a significant interaction between placenta accreta with age and anaemia status, although no significant interaction was found in twin pregnancies, suggesting different patterns of harm from placenta accreta in twin *vs*. singleton pregnancies.

Consistent with the study by Guo et al. [14], our research found that the incidence of ART is higher in twin pregnancies, and twin pregnancies are more likely to be complicated by PE and ICP. However, previous studies have been inconclusive regarding the proportion of twin pregnancies complicated by placenta accreta and concurrent placenta previa, reporting both higher and lower proportions [13,14,43]. In our study, no significant difference was found. Notably, we discovered that the occurrence of placenta previa with bleeding and racquet placenta was significantly higher in women with twin pregnancies complicated by placenta accreta. Although Guo's research indicates no significant difference in perioperative events between multiple and singleton pregnancies among patients with placenta accreta [14], our study, along with that of Miller et al. [13], shows that the risk of adverse perinatal outcomes is higher in twin pregnancies complicated by placenta accreta. This discrepancy in results may stem from differences in study design and the outcomes analysed. Guo’s study [14], while comparing several specific pregnancy outcomes, focused on a narrower set of outcomes and did not adequately exclude the influence of multiple pregnancies, which could have confounded the results. In contrast, while Miller et al. found significant differences, their analysis primarily used the severe maternal morbidity (SMM) criteria set by the CDC to evaluate mixed complications [13]. However, their study lacked a detailed examination of individual complications, limiting the depth and specificity of the findings. In our study, we compared a broader range of pregnancy complications and focused specifically on placenta-related complications, which provided a more detailed and targeted analysis. This approach allowed us to more reliably assess the differences in outcomes between singleton and twin pregnancies with placenta accreta, thereby strengthening the validity of our results. Our study revealing that in twin pregnancies with placenta accreta, the risks of premature delivery, caesarean section, pelvic inflammation, atonic postpartum haemorrhage, and premature rupture of membranes are 6.77, 2.39, 2.54, 5.84, and 2.93 times higher, respectively, than in singleton pregnancies with placenta accreta. These results are likely linked to the increased placental size in twin pregnancies. In twin pregnancies, the placental area is significantly enlarged, and when combined with placenta accreta, especially invasive or penetrating types, it leads to extensive uterine myometrial damage. This, in turn, increases surgical difficulty and the risk of haemorrhage [44,45]. The enlarged placental volume also exerts pressure on blood vessels, and the sudden release of this pressure after placental delivery destabilises haemodynamics, thereby raising the risk of postpartum haemorrhage [45]. Additionally, twin pregnancies are associated with increased uterine blood flow, which exacerbates placenta accreta-induced spiral artery remodelling. This disruption leads to abnormal vascular proliferation, impaired angiogenesis, and compromised tissue oxygenation, all of which further increase the risk of bleeding [46]. Moreover, excessive uterine expansion in twin pregnancies results in myometrial thinning, making the uterus more vulnerable to penetrating haemorrhage and uterine atony. This increases the difficulty of vaginal delivery and the likelihood of caesarean section [50]. Finally, the increased angiogenesis in twin pregnancies, particularly when combined with placenta accreta, enhances placental adhesion to surrounding tissues. This complicates placental separation and further raises the incidence of caesarean section [1]. This finding underscores the need for increased attention and management of placenta accreta in twin pregnancies within clinical practice, particularly given the 6.77-fold higher risk of preterm birth and the 5.84-fold higher risk of atonic postpartum haemorrhage compared to singletons. These striking figures suggest that clinicians must be particularly vigilant in monitoring and managing twin pregnancies with placenta accreta to prevent and mitigate these adverse outcomes. Additionally, the significant differences observed suggest that further research is needed to explore the specific mechanisms by which placenta accreta impacts singleton *vs*. twin pregnancies, and to develop targeted strategies for effectively managing these risk factors within current obstetric management standards. However, since this study aimed to explore additional secondary pregnancy outcomes, we did not apply correction for multiple testing. All analyses were exploratory and consistent with previous research methods. This may increase the risk of obtaining low *P*-values by chance, and therefore, the results should be interpreted with caution [13,14].

The mediation analysis confirmed that placenta accreta mediates the impact of twin pregnancies on adverse perinatal outcomes. For caesarean section, twin pregnancies had a total effect of 0.144 (95% CI = 0.115, 0.172, *P* < 0.001), with a direct effect of 0.135 and an indirect effect through placenta accreta of 0.091 (63.19% mediation). The effect of twin pregnancies on caesarean section is multifaceted, with both direct influences on the determinants of caesarean section and indirect effects through the impact on placenta accreta. This suggests that the determinants of caesarean section may involve multiple layers, including the direct physiological or pathological factors of twin pregnancies, such as a higher risk of complications including malpresentation, preterm birth, and preeclampsia [49], which often necessitate resolution via caesarean section. Additionally, there are indirect factors represented by placenta accreta. For postpartum haemorrhage, twin pregnancies showed no direct effect, but the indirect effect through placenta accreta was 0.014 (95% CI = 0.006, 0.024, *P* < 0.001). Twin pregnancies do not directly increase the risk of its occurrence but do so indirectly by affecting placenta accreta. This may be related to the increased area of the uterine lining compressed by the twin pregnancy, which may prompt a deeper implantation of the placenta into the myometrium, increasing the incidence of abnormal placental implantation. Consequently, the natural detachment of the placenta after delivery may be obstructed, leading to an inability of the uterus to contract and close blood vessels normally, thereby failing to effectively stop bleeding and ultimately increasing the risk of postpartum haemorrhage.

This study deepens our understanding of the significant impact of placenta accreta in twin pregnancies, particularly in increasing the risks of caesarean section, postpartum haemorrhage, and preterm birth. Given the higher risks in twin pregnancies, early prenatal detection of the placenta accreta is essential. Advanced imaging techniques like 3D ultrasound or MRI can help identify high-risk cases early, enabling more precise management and personalised care. A multidisciplinary team approach is critical for these pregnancies. Early collaboration among obstetricians, maternal-foetal medicine specialists, radiologists, and anaesthesiologists ensures timely interventions, such as planned caesarean section or postpartum haemorrhage management, improving both maternal and neonatal outcomes. Diagnosing placenta accreta in twin pregnancies is challenging, especially when the placenta is posterior or obscured by foetal parts. This highlights the need for expert prenatal screening. Early detection and intervention can reduce the risk of complications and severe outcomes, including hysterectomy and maternal mortality. Thus, establishing clinical guidelines and advancing multidisciplinary care for twin pregnancies with placenta accreta should be a priority. Standardised protocols can enhance care consistency and improve clinical outcomes, particularly in resource-limited settings.

### Strength and limitation

The strengths of our study include the utilisation of a multi-year database from Women and Children’s Hospital of Chongqing Medical University, a tertiary teaching hospital in Southwest China, ensuring the richness and continuity of the research data. Our cases were derived from strict pathological or clinical diagnoses, rather than relying solely on ICD coding, which substantially ensured the accuracy of the diagnoses of our study subjects. A consistent medical team managed patients at the same centre with standardised surgical strategies and preoperative, intraoperative, and postoperative management protocols, reducing potential outcome biases that could arise from different caregivers. The study also meticulously recorded common pregnancy complications and placental information, including anaemia, hypothyroidism, GDM, PE, ICP, and FGR, as well as placental morphologies such as racquet-shaped placenta and sail-shaped placenta, and various conditions of placenta previa, including placenta previa with bleeding, central placenta previa, marginal placenta previa, and low-lying placenta, all of which provided more detailed and comprehensive data support.

However, the limitations of our study should not be overlooked. Due to its retrospective nature, we were unable to obtain or quantify potential confounding factors such as dietary habits, physical activity levels, and mental health status of pregnant women, which may limit our comprehensive understanding of the impact of placenta accreta. Additionally, despite collecting extensive data on placenta accreta spectrum disorders, we could not ascertain the specific timing of diagnosis between singleton and twin cohorts, nor confirm the exact number of previous caesarean deliveries for the patients, which had to be considered as a potential confounding factor. For example, prenatal identification of high-risk placenta accreta patients can allow for tailored delivery plans at medical centres equipped with the necessary surgical expertise, thus reducing the risk of adverse outcomes. This study focused only on cases of placenta accreta within the PAS, and did not record other severe complications such as transfusion, hysterectomy, amniotic fluid embolism, or disseminated intravascular coagulation, although we selected common and significant adverse perinatal outcomes such as postpartum haemorrhage, caesarean section, premature rupture of membranes, and uterine rupture, which all provided valuable information for our understanding of the impact of placenta accreta on the health of women with twin and singleton pregnancies. This research lacks specific information regarding whether patients received selective interventions or emergency interventions. It is noteworthy that emergency delivery may pose more complex situations for up to 30% of patients with placenta accreta and is associated with poorer maternal outcomes. Lastly, although the RCS model provides meaningful insights into the nonlinear relationship between gestational weeks at delivery and the risks of caesarean section and postpartum haemorrhage in both singleton and twin pregnancies, with and without placenta accreta, there are certain limitations in visualising the risk changes. Due to the limited number of twin pregnancies delivering after 39 weeks and the insufficient sample size to perform risk stratification for each gestational week, it is not possible to accurately calculate the risks for each week, and the confidence intervals may be too wide. Therefore, while the current analysis provides valuable information, future studies should increase the sample size, particularly for twin pregnancies, to further enhance the precision of risk assessment. In addition, currently available statistical tools (such as the mediation package) do not support sensitivity analysis when the outcome, mediator, and independent variables are all binary. This limits our ability to comprehensively assess the impact of potential unmeasured confounders. Future research should explore sensitivity analysis methods suitable for this situation to further enhance the robustness of causal inferences.

## CONCLUSIONS

This study highlights that twin pregnancies are significantly associated with an increased risk of placenta accreta and subsequent adverse perinatal outcomes compared to singleton pregnancies. The findings underscore the importance of early diagnosis and careful monitoring of twin pregnancies, especially those with placenta accreta, to improve perinatal outcomes. Antenatal interventions and tailored delivery strategies are crucial in managing these high-risk pregnancies, particularly in preventing complications such as preterm birth, caesarean section, and postpartum haemorrhage. Future research should focus on refining management guidelines and exploring potential preventive measures for these at-risk pregnancies.

## Additional material


Online Supplementary Document
