# Supplementary material for: Differential risk and clinical characteristics of placenta accreta spectrum in twin and singleton pregnancies: implications for perinatal outcomes
Source: J Glob Health. 2025 Aug 22;15:04252. doi: 10.7189/jogh.15.04252 (PMC12371300; doi:10.7189/jogh.15.04252)

Supplement to: Wei-Zhen T, Kang-Jin H, Xia L, Qin-Yu C, Ying-Xiong W, Hong-Yu X, Li W, Lan W, Tai-Hang L. Differential risk and clinical characteristics of placenta accreta spectrum in twin and singleton pregnancies: implications for perinatal outcomes. J Glob Health. 2025;15:04252.

Table S1. The association between twin pregnancy and placenta accreta.

	1:1 PSM	P.value	1:2 PSM	P.value	1:3 PSM	P.value
Placenta accreta	1.330(1.049,1.689)	0.019*	1.520(1.236,1.869)	<0.001*	1.652(1.360,2.006)	<0.001*
Placenta accreta with bleeding	1.675(1.031,2.776)	0.040*	2.245(1.448,3.480)	<0.001*	2.580(1.711,3.890)	<0.001*

*$p < 0.05$

Table S2. Demographic and clinical characteristics of placenta accreta occurrence in women with twin and singleton pregnancies.

Characteristic [N (%)]	Twin pregnancy				Singleton pregnancy			
	Placenta accreta	Non-Placenta accreta	Statistic ($\chi2$)	*P*.value	Placenta accreta	Non-Placenta accreta	Statistic ($\chi2$)	*P*.value
Age (Years)	32.42[29.74,34.58]	30.88[28.46,33.32]	-4.512	<0.001*	32.26[28.87,35.07]	30.46[27.51,33.77]	-10.853	<0.001*
PBMI, M (Q_1, Q_3)	21.26[20.03,23.44]	21.51[20.06,23.53]	0.924	0.356	21.94[19.98,24.35]	21.10[19.03,23.63]	-7.614	<0.001*
ART, n(%)	60(34.29)	238(20.33)	17.215	<0.001*	55(4.58)	243(1.69)	49.100	<0.001*
Nulliparity, n (%)	154(88.00)	1008(86.08)	0.475	0.490	702(58.40)	9205(64.10)	15.572	<0.001*
Primigravida, n (%)	70(40.00)	632(53.97)	11.909	<0.001*	256(21.30)	5088(35.43)	98.269	<0.001*
Age of first menstruation	12.00[11.00,13.00]	12.00[11.00,13.00]	-0.164	0.864	12.00[11.00,13.00]	12.00[11.00,13.00]	0.452	0.635
History of cesarean section, n(%)	0(0.00)	3(0.26)	nan	nan	53(4.41)	527(3.67)	1.690	0.194
History of smoking, n(%)	4(2.29)	22(1.89)	0.126	0.722	43(3.61)	387(2.71)	3.262	0.071
History of alcoholism, n(%)	37(21.39)	144(12.43)	10.30	0.001*	201(17.09)	2262(15.96)	1.024	0.312
Anaemia, n(%)	9(5.14)	194(16.57)	15.515	<0.001*	89(7.40)	1332(9.28)	4.682	0.030*
Hypothyroidism, n(%)	11(6.29)	54(4.61)	0.929	0.335	148(12.31)	2127(14.81)	5.550	0.018*
GDM, n(%)	69(39.43)	424(36.21)	0.680	0.410	409(34.03)	5227(36.40)	2.704	0.100
PE, n(%)	36(20.57)	217(18.53)	0.415	0.519	79(6.57)	548(3.82)	21.791	<0.001*

FGR, n(%)	5(2.86)	98(8.37)	6.545	0.011*	16(1.33)	199(1.39)	0.024	0.876
ICP, n(%)	30(17.14)	198(16.91)	0.006	0.939	38(3.16)	573(3.99)	2.020	0.155
Racquet placenta, n(%)	6(3.43)	55(4.70)	0.566	0.452	41(3.41)	438(3.05)	0.484	0.487
Velaria placenta, n(%)	8(4.57)	31(2.65)	2.003	0.157	19(1.58)	91(0.63)	14.172	<0.001*
Placenta previa, n(%)	18(10.29)	25(2.14)	32.706	<0.001*	144(11.98)	252(1.76)	467.626	<0.001*
Placenta previa with bleeding, n(%)	10(5.71)	11(0.94)	22.601	<0.001*	27(2.25)	96(0.67)	35.210	<0.001*
Central placenta previa , n(%)	14(8.00)	7(0.60)	54.316	<0.001*	68(5.66)	110(0.77)	234.677	<0.001*
Marginal placenta previa, n(%)	4(2.29)	14(1.20)	1.371	0.242	59(4.91)	111(0.77)	175.565	<0.001*

Abbreviation: PBMI, Pre-pregnancy body mass Index; ART, Assisted reproductive technology; GDM, Gestational diabetes mellitus; PE, Preeclampsia; FGR, Fetal growth restriction; ICP, Intrahepatic cholestasis of pregnancy. $*p < 0.05$

Table S3. Stratified and interaction analysis of placenta accreta-associated adverse perinatal outcomes in twin and singleton pregnancies.

	**Twin pregnancy**						**Singleton pregnancy**					
	**Cesarean section**			**Postpartum hemorrhage**			**Cesarean section**			**Postpartum hemorrhage**		
	**aOR(95%CI)**	***P*-value**	***P*-interaction**	**aOR(95%CI)**	***P*-value**	***P*-interaction**	**aOR(95%CI)**	***P*-value**	***P*-interaction**	**aOR(95%CI)**	***P*-value**	***P*-interaction**
Age			0.026*			0.357			0.188			0.021*
≤35	3.29(1.99, 5.46)	<0.001*		3.13(1.44,6.8)	0.004*		2.65(2.29,3.06)	<0.001*		2.75(2.09,3.62)	<0.001*	
>35	22.55(5.06,100.57)	<0.001*		5.91(1.69,20.64)	0.005*		3.24(2.39,4.39)	<0.001*		1.29(0.64,2.6)	0.482	
ART			0.027*			0.751			0.013			0.154
No	5.55(3.24, 9.51)	<0.001*		3.33(1.56,7.14)	0.002*		2.67(2.34,3.05)	<0.001*		2.5(1.92, 3.26)	<0.001*	
Yes	1.68(0.62, 4.59)	0.308		5.27(1.54,17.96)	0.008*		9.22(3.42,24.86)	<0.001*		1.4(0.52, 3.77)	0.507	
History of cesarean section			NA			NA			0.997			0.112
No	4.60(2.85,	<0.		3.78(2,7.	<0.		2.83(2.4	<0.		2.31(1.7	<0.	

	7.41)	001*		16)	001*		8,3.23)	001*		8,3)	001*	
Yes	NA	NA		NA	NA		NA	NA		10.68(2.09,54.5)	0.004*	
Placenta previa			0.967			0.986			0.001*			0.366
No	4.81(2.85, 8.11)	<0.001*		3.97(2.06,7.66)	<0.001*		2.49(2.17,2.86)	<0.001*		2.18(1.64,2.91)	<0.001*	
Yes	5.32(1.07, 26.5)	0.042*		NA	NA		5.97(3.74,9.55)	<0.001*		1.42(0.74,2.74)	0.293	
Anaemia			0.588			0.986			0.064			<0.001*
No	4.36(2.66, 7.13)	<0.001*		4.26(2.19,8.26)	<0.001*		2.85(2.49,3.27)	<0.001*		2.06(1.57,2.72)	<0.001*	
Yes	7.34(0.87, 61.79)	0.067		NA	NA		1.93(1.23,3.04)	0.004*		8.85(4.15,18.86)	<0.001*	
Hypothyroidism			0.715			0.987			0.387			0.575
No	4.44(2.72, 7.27)	<0.001*		3.99(2.08,7.64)	<0.001*		2.82(2.45,3.25)	<0.001*		2.25(1.71,2.98)	<0.001*	
Yes	7.49(0.87, 64.26)	0.066		NA	NA		2.38(1.67,3.39)	<0.001*		3.04(1.55,5.96)	0.001*	
PE			0.899			0.057			0.389			0.984
No	4.52(2.71, 7.54)	<0.001*		5.68(2.72,11.89)	<0.001*		2.7(2.36, 3.09)	<0.001*		2.37(1.82,3.09)	<0.001*	
Yes	5.73(1.28, 25.67)	0.022*		0.89(0.17,4.62)	0.889		3.53(1.75,7.12)	<0.001*		2.13(0.79,5.72)	0.135	
ICP			0.001*			0.669			0.256			0.523
No	7.12(3.91, 12.97)	<0.001*		4.18(2.11,8.26)	<0.001*		2.72(2.38,3.11)	<0.001*		2.35(1.81,3.05)	<0.001*	
Yes	0.79(0.3,2.04)	0.621		0.89(0.11,6.88)	0.910		4.38(1.99,9.67)	<0.001*		3.48(0.92,13.11)	0.066	

Abbreviation: CI: confidence interval, SD : standard deviation; ART, Assisted reproductive technology; GDM, Gestational diabetes mellitus; PE, Preeclampsia; ICP, Intrahepatic cholestasis of pregnancy.

aOR adjusted for maternal age, PBMI, Nulliparity, Primigravida, the history of cesarean section, History of alcoholism, Racquet placenta, Velaria placenta, placenta previa, GDM, PE, ICP, FGR, ART. **p* < 0.05.

Table S4. Clinical characteristics of women with placenta accreta in twin versus singleton pregnancies.

Characteristic [N (%)]	Twin pregnancy (n = 175)	Singleton pregnancy (n = 1,202)	Statistic ($\chi 2$)	P.value
Uterine myoma, n(%)	12(6.86)	57(4.74)	1.436	0.231
Anaemia, n(%)	9(5.14)	89(7.40)	1.182	0.277
Hypothyroidism, n(%)	17(9.71)	148(12.31)	0.978	0.323
GDM, n(%)	69(39.43)	409(34.03)	1.967	0.161
PE, n(%)	36(20.57)	79(6.57)	39.113	<0.001*
FGR, n(%)	5(2.86)	16(1.33)	2.369	0.124
ICP, n(%)	30(17.14)	38(3.16)	63.611	<0.001*
Racquet placenta, n(%)	6(3.43)	41(3.41)	0.000	0.990
Velaria placenta, n(%)	8(4.57)	19(1.58)	7.108	0.008*
Placenta previa, n(%)	18(10.29)	144(11.98)	0.422	0.516

Placenta previa with bleeding, n(%)	10(5.71)	27(2.25)	7.026	0.008*
Central placenta previa , n(%)	14(8.00)	68(5.66)	1.497	0.221
Marginal placenta previa, n(%)	4(2.29)	59(4.91)	2.407	0.121
Placenta hypoposition, n(%)	4(2.29)	28(2.33)	0.001	0.971

Abbreviation: PBMI, Pre-pregnancy body mass Index; GDM, Gestational diabetes mellitus; PE, Preeclampsia; FGR, Fetal growth restriction; ICP, Intrahepatic cholestasis of pregnancy. *$p < 0.05$

Table S5. Comparison of adverse perinatal outcomes associated with twin and singleton pregnancies in patients with placenta accreta.

	**Twin pregnancy (n = 175)**	**Singleton pregnancy (n = 1,202)**	**p.value**	**Univariate analysis**	**Multivariate analysis**
Premature delivery	60(34.29)	88(7.32)	<0.001*	6.61(4.52,9.66)	6.77(4.41,10.39)
Cesarean section	153(87.43)	837(69.63)	<0.001*	3.03(1.91,4.82)	2.39(1.46,3.91)
Pelvic inflammation	50(28.57)	143(11.90)	<0.001*	2.96(2.04,4.30)	2.54(1.68,3.83)
Postpartum hemorrhage	17(9.71)	77(6.41)	0.105	1.57(0.91,2.73)	1.31(0.70,2.43)
Atonic postpartum hemorrhage	12(6.86)	16(1.33)	<0.001*	5.46(2.54,11.74)	5.84(2.41,14.17)
Premature rupture of membranes	20(11.43)	45(3.74)	<0.001*	3.32(1.91,5.77)	2.93(1.60,5.37)
Uterine rupture	3(1.71)	26(2.16)	0.699	0.79(0.24,2.63)	2.01(0.51,7.94)
Umbilical entanglement	36(20.57)	308(25.62)	0.149	0.75(0.51,1.11)	0.71(0.47,1.08)
Fetal distress	6(3.43)	130(10.82)	0.002*	0.29(0.13,0.67)	0.22(0.09,0.52)

aOR adjusted for maternal age, PBMI, Nulliparity, Primigravida, History of cesarean section, History of alcoholism, Racquet placenta, Velaria placenta, Placenta previa, GDM, PE, ICP, FGR, ART.

Abbreviation: PBMI – pre-pregnancy body mass index, GDM – gestational diabetes mellitus, PE – preeclampsia, ICP – intrahepatic cholestasis of pregnancy, FGR – foetal growth restriction, ART – assisted reproductive technology.

*$p < 0.05$.

Table S6. Mediation analysis of placenta accreta in the association between twin pregnancy and cesarean section outcomes.

	**Coefficient**	**S.E.**	***p*-value**	**CI [2.5%]**	**CI [97.5%]**

Exposure-mediator effect	0.361	0.094	<0.001*	0.176	0.546
Mediator-outcome effect	0.257	0.014	<0.001*	0.230	0.283
Total effect	0.144	0.014	<0.001*	0.115	0.172
Direct effect	0.135	0.014	<0.001*	0.107	0.163
Indirect effect	0.091	0.025	<0.001*	0.043	0.137

Abbreviation: S.E., Standard Error. $*p < 0.05$

Table S7. Mediation Analysis of the Impact of Placenta Accreta on the Incidence of Postpartum Hemorrhage in Twin Pregnancies.

	**Coefficient**	**S.E.**	***p*-value**	**CI [2.5%]**	**CI [97.5%]**
Exposure-mediator effect	0.361	0.094	<0.001*	0.176	0.546
Mediator-outcome effect	0.039	0.005	<0.001*	0.029	0.048
Total effect	-0.004	0.005	0.411	-0.014	0.006
Direct effect	-0.006	0.005	0.285	-0.016	0.005
Indirect effect	0.014	0.005	<0.001*	0.006	0.024

Abbreviation: S.E., Standard Error. $*p < 0.05$

Figure legends

Figure S1 Causal diagram representing simple mediation. Legend: X: the exposure, M: the mediator, Y: the outcome, C: a set of confounders.

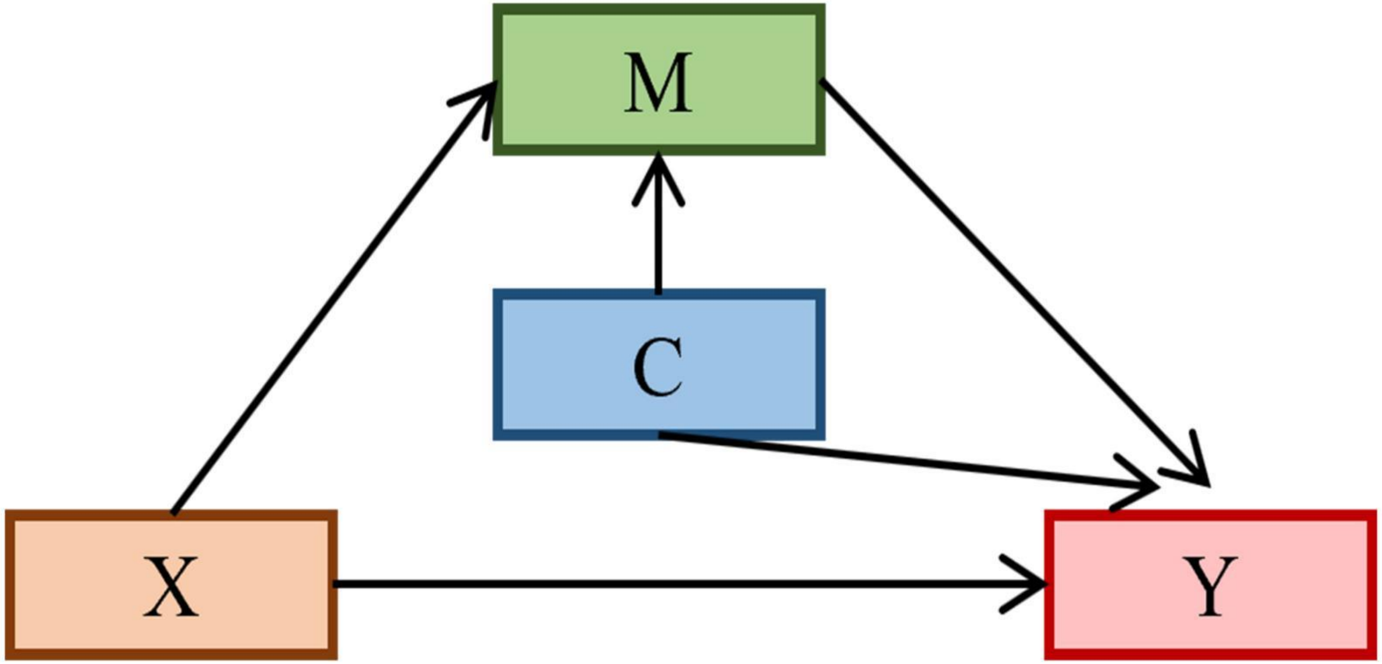

Supplement: Online Supplementary Document [file jogh-15-04252-s001.pdf]
